# Long term patterns and risk factors of loneliness in young adults from an 18-Year longitudinal study in Germany

**DOI:** 10.1038/s41598-025-08842-1

**Published:** 2025-07-05

**Authors:** Max Supke, Kurt Hahlweg, Ann-Katrin Job, Wolfgang Schulz

**Affiliations:** 1 Institute of Psychology, Department of Clinical Psychology, Psychotherapy and Diagnostics, Technische Universität Braunschweig, Humboldtstr. 33, 38106 Braunschweig, Germany; 2 Leibniz Institute for Resilience Research (LIR), Mainz, Germany; 3 Institute of Psychology, Universität Kassel, Kassel, Germany

**Keywords:** Emotional loneliness, Young adults, Protective and risk factors, Longitudinal study, Prevention, Risk factors, Psychology

## Abstract

Preventing loneliness is a significant goal due to its association with long-term negative effects on both mental and physical health. This German longitudinal study used three assessment points to examine predictors of emotional loneliness—defined as the absence of close, trusting relationships—in young adulthood (T3: age 22), based on factors assessed in early childhood (T1: age 4) and adolescence (T2: age 14). Utilizing data from 224 families, regression models were employed to identify child and parent risk factors—such as child and parent mental health problems, parent dysfunctional parenting, child adverse childhood experiences, child problematic internet use, and child experiences of school bullying—to predict child emotional loneliness. Emotional loneliness was measured through self-reports by adolescents/young adults and external reports provided by parents. Over 25% of adolescents reported emotional loneliness, a figure that increased to 50% in young adulthood during the pandemic. Experiencing loneliness in adolescence was associated with a significantly higher likelihood of feeling lonely again in emerging adulthood. Parents tend to underestimate their children’s feelings of loneliness. Lower levels of childhood externalizing mental health problems and higher levels of maternal dysfunctional parenting were associated with an increased likelihood of loneliness in adolescence. Only adolescent mental health problems predicted loneliness in emerging adulthood. Additionally, more adverse childhood experiences and higher levels of compulsive internet use during adolescence were linked to more adolescent mental health problems, identifying potential indirect targets for the prevention of loneliness. In contrast, paternal factors did not significantly contribute to the prediction of emotional loneliness in this sample. Effective strategies may include enhancing parenting practices and increasing awareness of child mental health problems, mitigating adverse childhood experiences, and reducing excessive internet use.

## Introduction

For a long time, loneliness was primarily considered a problem affecting older adults, particularly the elderly. However, over the past decade, and especially since the COVID-19 pandemic, increasing attention has been directed toward other demographic groups, including *young adults*^1^. This demographic group was previously assumed to be very active in their free time, with numerous social connections. However, societal changes, such as the rise of social media^2^, and the shift toward more remote work environments^3^, may have contributed to altered experiences of loneliness, particularly among young people. Furthermore, young adults undergoing transitional phases, such as entering the workforce or pursuing higher education, represent a particularly vulnerable group that faces numerous challenges. These transitions often involve significant life changes, such as relocating, forming new social networks, or managing an independent household. While some individuals adapt successfully to these changes, others may struggle, which can increase their risk of experiencing loneliness^4^.

Loneliness is defined in various ways^5^, with the most frequently cited definition in the literature being that of Perlman and Peplau^6^. According to their definition, loneliness is a negative emotional state associated with the perception that one’s social relationships are inadequate in quantity or quality. Furthermore, a distinction is made between emotional and social loneliness. *Emotional loneliness* refers to the absence of close, trusting relationships, while *social loneliness* refers to the absence of a broader social network of friends and acquaintances. Additionally, distinctions are made between episodic and chronic loneliness^7^, as well as between the risk of becoming lonely and the risk of remaining lonely^8^. Chronic feelings of loneliness have been shown to alter the nature and likelihood of social interactions and increase the activity of stress pathways through the activation of the autonomic nervous system and the hypothalamic-pituitary-adrenocortico axis^9^.

Kirwan et al.^10^ identified a significant increase in published studies on loneliness among young adults since 2016 (2000–2015: 112 studies; 2016–2021: 201 studies). Despite this growth in research, knowledge about this group remains relatively limited, particularly in Germany, where few studies address prevalence rates, risk factors, and consequences of loneliness. This knowledge gap is largely due to the predominance of cross-sectional studies using convenience samples^10^. Only empirical data from population-based longitudinal studies can provide the foundation necessary to develop targeted and effective preventive interventions.

### Prevalence of loneliness

*Epidemiological studies* suggest that young adults aged 18 to 25 and individuals over 65 are most frequently affected by loneliness, with loneliness following a U-shaped distribution^9,11^. However, Barjaková et al.^11^ caution that age alone is not decisive; rather, the varying prevalence rates of loneliness across different age groups may be explained by differences in the prevalence of specific risk factors for loneliness.

Depending on the definition and measurement of loneliness, a wide range of prevalence rates has been reported. Surkalim et al.^12^ conducted a meta-analysis of loneliness prevalence rates across 113 countries, drawing from 57 studies published between 2000 and 2019. This meta-analysis further differentiated between several age groups, with data available for 18- to 29-year-olds from 30 countries. Loneliness was measured using both scales and single-item questions. The lowest prevalence rates of loneliness based on single items were consistently observed in Northern European countries (1.8% to 4.5%) and the highest in Eastern European countries (7.5% to 94%), with Germany at 5.0%. Although somewhat outdated, these data highlight cross-cultural differences, showing that loneliness is highly culture-dependent. This limits the generalizability of findings across countries.

More recent studies report higher prevalence rates. For example, a study conducted in Britain found that 23 to 31% of young adults reported feeling “sometimes” lonely, while 5% to 7% reported feeling “often” lonely^13^. Witters^14^ repeatedly surveyed young adults (age under 30) in the United States between March 2022 and February 2023 and found a decline in loneliness, with a final rate of 24%.

Several studies in Germany have also begun to explore the prevalence rates of loneliness among young adults. For instance, Entringer^15^ reported that 14% of individuals under 30 experienced loneliness at least occasionally. In a post-pandemic survey of 16- to 20-year-olds, Luhmann et al.^16^ found that 16.3% to 18.5% of respondents reported significant loneliness, while 51.2% to 78.0% experienced moderate loneliness. Similarly, in the study “Extremely Lonely?” 55% of respondents aged 16 to 23 indicated that they sometimes or always missed companionship, and 26% felt a lack of closeness to others^17^. These prevalence rates suggest that feelings of loneliness are not uncommon among young adults; they occur with notable frequency.

Numerous studies have confirmed that feelings of loneliness among young people have increased since the COVID-19 pandemic (e.g.,^18^). However, less well-known is the fact that an *upward trend* in loneliness among young adults was evident even before the pandemic (between 1976 and 2019;^19^).

### Long-term consequences of loneliness

The need for both curative and, more critically, preventive interventions becomes evident when considering the *significant health and economic consequences* of loneliness in young adulthood (ages 18 to 25). In their meta-analysis of 114 studies across various age groups, Park et al.^20^ concluded that loneliness has moderate to large effects on several health outcomes, with the most pronounced effects observed in mental health, particularly in relation to suicidality, depression, and overall mental well-being. Loneliness is a significant predictor of both suicidal ideation and behavior across all age groups^21^.

Longitudinal studies also reveal lasting associations between loneliness, economic outcomes, and mental health. A Norwegian study^22^ showed that adolescents with elevated loneliness, and those whose loneliness increased into young adulthood, were more likely to be prescribed psychotropic medications 15 years later in adulthood. The authors concluded that early loneliness increases the risk of developing severe mental disorders later. Another longitudinal study by Bryan et al.^23^ found that children who were lonelier at age 12 were more likely to be not in employment, education, or training and rated their employability and subjective social status lower in young adulthood. The authors concluded that loneliness could have direct economic costs, resulting from reduced employability and lower social status. According to a study by Xerxa et al.^24^, childhood loneliness was associated with anxiety symptoms and depressive disorders in young adulthood, indicating that loneliness—even in childhood—could have long-term mental health consequences. These findings underscore the importance of early interventions to prevent loneliness and mitigate its effects.

### Risk factors for loneliness

Lim and colleagues^7^ developed a *model of loneliness* that is relevant across age groups. This model distinguishes between (a) triggers, (b) risk factors and correlates, (c) loneliness as a consequence, and (d) solutions. Triggers include, for example, critical life events. Relevant risk factors and correlates are categorized into demographic data (e.g., marital status), health factors (e.g., physical and mental health), and socio-environmental factors (e.g., digital environment, workplace). The authors^7^ emphasize that there are multiple interactions between these variables, and that bidirectional influences must be considered. This model allows for the systematization of risk factors and correlates, while also highlighting potential curative and preventive interventions.

While a wealth of studies has examined risk factors in adults^11^, particularly among the elderly^25^, it remains unclear whether these findings can be generalized to young adults. Significantly fewer studies have specifically investigated risk factors in young adulthood. A scoping review^10^ could show that family and social relationship factors, as well as mental health, are among the most frequently examined potential risk and protective factors. Botha and Bower^26^ conducted a longitudinal study of Australian men aged 15 to 98. For the 15- to 24-year-old age group, they identified the following significant risk factors for loneliness: lower job security, life events such as the death of a close friend or separation from a spouse or partner, low frequency of social contacts, lower neighborhood satisfaction, and long-term disability.

Lonely young adults were more likely to have experienced mental health problems during childhood and to have been subjected to bullying and social isolation^13^. Moreover, childhood bullying victimization has been found to predict loneliness in young adulthood, even if the victimization did not persist^27^. Adverse childhood experiences (ACEs), such as neglect or abuse, are associated with worse mental and physical health outcomes across the life span^28^. Emotional neglect and cumulative ACEs were found to be positively associated with greater loneliness in emerging adulthood^29^. Higher social media use is associated with increased feelings of social isolation^30^ and loneliness^2^. Far less is known about the relationship between childhood parenting experiences and later feelings of loneliness. There is some evidence suggesting that harsh parenting may be associated with increased feelings of loneliness^31^.

### Current research gaps

Existing research on loneliness among young adults reveals *significant research gaps and methodological deficiencies*, which have implications for future research. In their comprehensive review of 201 studies on loneliness in young adults, Kirwan et al.^10^ conclude that the literature is dominated by cross-sectional studies with convenience samples. They emphasize the need for more population-based longitudinal research to understand the factors that predict loneliness over time and the subsequent effects of loneliness on young people (see above). In addition, the following key weaknesses in the current research can be identified:

- Although there are now over 300 studies on loneliness among young adults, knowledge about this group remains relatively limited compared to adult and older populations.
- Representativeness is further limited by the fact that most studies focus on university students.
- The majority of studies originate from English-speaking countries. While some studies have been conducted in Germany in recent years, the volume is relatively low compared to the United States, the United Kingdom, and Australia.
- While the consequences of loneliness are well-studied, and many risk factors and correlates have been identified, there is a lack of research on early childhood risk factors.
- There is a notable lack of studies focusing on the prevention of loneliness.

### The present study

The present longitudinal study, which includes data on emotional loneliness collected over an 18-year period, aims to address some of these gaps. The following research questions are examined:

1. *Prevalence:* How frequently does emotional loneliness occur among young adults?
2. *Stability:* How stable are feelings of loneliness over time? Have feelings of loneliness changed from adolescence to young adulthood? What proportion of individuals experience chronic loneliness?
3. *Parent–child agreement:* Are parents able to accurately report on the extent of their children’s feelings of emotional loneliness?
4. *Risk factors:* Which risk factors across childhood (increased child mental health problems, more dysfunctional parenting, increased parental mental health problems, higher exposure to adverse childhood experiences) and adolescence (increased mental health problems, higher exposure to adverse childhood experiences, experiences of school bullying, higher problematic internet use) are associated with emotional loneliness in young adulthood?

The analyses are conducted from both maternal and paternal perspectives, because research indicates that fathers interact with their children, perceive them, and maintain relationships with them in ways that differ from mothers (e.g.,^32,33^). Thus it is important to not only include mothers’ but also fathers’ perspectives for a comprehensive understanding of child psychopathology.

## Methods

### Original sample recruitment

The “Future Family” (FF) project, which began in 2001/2002, consists of two sub-studies. The FF I-study^34^ was a randomized controlled trial, while the FF II-study^35^ was an open trial without a control group. The primary reason for conducting the FF II-study was the underrepresentation of families from lower socioeconomic backgrounds in the FF I-study. Both studies aimed to evaluate the effectiveness of the Positive Parenting Program (Triple P;^36^). Following these, the FF III-study conducted a 10-year follow-up (FU10;^37^), and the FF IV-study carried out an 18-year follow-up (FU18) of the FF I- and FF II-sample. These follow-up studies used a longitudinal design to examine the lasting effectiveness of the Triple P and to predict mental health outcomes as participants transitioned into adolescence (14 years; FF III) and emerging adulthood (22 years; FF IV). The analyses included various risk and protective factors identified during the participants’ kindergarten years.

During the initial assessment (Pre), families with children aged 2.5 to 6 years were recruited from kindergartens in Braunschweig, a large city in Germany. The original sample included *N* = 477 families (FF I: *n* = 280 families, FF II: *n* = 197 families). At Pre, the average age of the mothers was *M* = 35.2 years (*SD* = 5.0), the fathers’ average age was *M* = 38.8 years (*SD* = 6.0), and the children’s average age was* M* = 4.1 years (*SD* = 1.0). A total of *n* = 458 families participated in the one-year follow-up (FU1; retention rate: 96%); *n* = 449 families participated in the two-year follow-up (FU2; 94.1%); *n* = 361 families continued to participate after 10 years (FU10; 75.7%), and *n* = 316 families participated in the 18-year follow-up (FU18; retention rate: 67.1%). Six of the original 477 families were excluded as they did not meet the inclusion criteria at FU18.

### Sample characteristics (FU18)

At FU18, the mean age of the emerging adults was 22.3 years (*SD* = 1.2), the mean age of the mothers was 53.5 years (*SD* = 4.8), and the mean age of the fathers was 56.5 years (*SD* = 4.9). The sex distribution among the emerging adults was nearly equal, with 48% being female and 52% male. In terms of their educational attainment, 10% of the emerging adults had no school leaving certificate or completed only 9 years of schooling, 18% had completed 10 years of schooling, and 73% had A-levels or high school education. Two-thirds (67%) of the families in the sample participated in the Triple P as part of the experimental condition, indicating that the analyzed data were derived from an intervention study sample. Regarding the parents’ educational background, 9% of mothers and 15% of fathers had no school leaving certificate or completed only 9 years of schooling, 37% of mothers and 24% of fathers had completed 10 years of schooling, and 55% of mothers and 61% of fathers had A-levels or high school education. Only 2% of the families were classified as having a low socioeconomic status (SES), 46% had a middle SES, and 52% had a high SES. About 19% of the families had a migration background.

There were significant sociodemographic differences at the pre-assessment between families who participated at FU18 and those who dropped out. Dropouts were more likely to be single parents (*p* < 0.001), had a lower SES (as indicated by the school leaving qualifications of the mothers and fathers, the Social Structure Index of their child’s kindergarten, and monthly household income; each *p* < 0.001), and were more likely to have a migration background (*p* = 0.036). Additionally, mothers who dropped out were significantly younger at baseline compared to those who participated at FU18 (*p* < 0.001) and reported significantly more symptoms of psychopathology at baseline (*p* = 0.027). Consequently, the representativeness of the FU18 sample is considerably limited when compared to the total baseline sample.

### Procedure

The Future Family surveys combined structured personal interviews, conducted separately with parents and emerging adults to assess sociodemographic data (e.g., SES, migration background), along with standardized questionnaires (e.g., on mental health problems, dysfunctional parenting). Up until FU10, these interviews were conducted in person during home visits. Due to the COVID-19 pandemic, at FU18, the assessments were primarily conducted via telephone and online through the SurveyMonkey platform (https://www.surveymonkey.de). To compensate for their time and participation in the approximately 2.5-h survey, both the young adults and parents received a financial compensation of 50 € each. Written informed consent was obtained from all participants prior to the survey, and the study adhered to the principles outlined in the Declaration of Helsinki. Ethical approval was granted by the ethics committee of the German Psychological Society (DGPs; identification number: WS 12_2010) and the independent ethics committee of Technische Universität Braunschweig (identification number: D-2019-01; Faculty of Life Sciences).

### Measures

The following instruments are presented in the order of their assessment:

#### Child mental health problems in kindergarten (Pre)

The Achenbach System of Empirically Based Assessment (ASEBA;^38^) was used to assess mental health problems longitudinally through parent reports using the Child Behavior Checklist (CBCL). Both mothers and fathers completed the checklist, which includes 110 items (e.g., “Cries a lot”), rating the frequency of problematic behaviors or emotional problems exhibited by their child on a 3-point Likert scale (0 = *not true*; 1 = *somewhat or sometimes true*; 2 = *very true or often true*). Scores were calculated for internalizing problems (e.g., symptoms of anxiety) and externalizing problems (e.g., aggressive behavior). Higher scores indicate greater mental health problems in the respective area. The internal consistencies were satisfactory at Pre, with α = 0.94 for mothers and α = 0.96 for fathers.

#### Parental dysfunctional parenting (Pre)

Dysfunctional parenting was assessed using the German version^39^ of the Parenting Scale (PS;^40^). Parents rated the usage of 35 parenting practices on a 7-point Likert scale (e.g., “When my child does something I don’t like… (1) …*I do something about it every time.*” to “(7) …*I just let it pass.*”). Higher scores indicate greater use of dysfunctional parenting practices, such as overreaction. The internal consistency was satisfactory in our sample, with α = 0.87 for mothers and α = 0.86 for fathers.

#### Parental mental health problems (Pre)

The German version of the Depression Anxiety Stress Scales (DASS) were used to assess symptoms of depression, anxiety, and stress (14 items allocated to each category, 42 total items) in mothers and fathers (^41^, German version:^42^). A higher score indicates higher mental distress in the last four weeks. The internal consistency in our sample was excellent (Pre: α mothers = 0.95/α fathers = 0.93).

#### Adolescent compulsive internet use (FU10)

The Compulsive Internet Use Scale (CIUS) is a questionnaire designed to measure internet addiction^43^. This self-report questionnaire assessed the severity of compulsive internet use using 14 items, each rated on a 5-point Likert scale (“How often do you find it difficult to stop using the internet when you are online?”—0 = *never*, 1 = *seldom*, 2 = *sometimes*, 3 = *often*, and 4 = *very often*). A higher score indicates more problematic internet usage. In our sample, the internal consistency was α = 0.88.

#### Adolescent mental health problems (FU10)

The Strengths and Difficulties Questionnaire for children (SDQ;^44^) is the German version of the Strengths and Difficulties Questionnaire by Goodman^45^. This self-report questionnaire assessed specific appropriate behaviors as well as behavioral and emotional problems in children and adolescents aged between four and sixteen years using 25 items (e.g., “Often has temper tantrums or hot tempers”—1 = *not true*, 2 = *somewhat true*, 3 = *certainly true)*. A higher score indicates more mental health problems in the last 6 month. The internal consistency was α = 0.72.

#### Emotional loneliness (FU10 and FU18)

The extent to which adolescents (Youth Self-Report; YSR) and emerging adults (Adult Self-Report; ASR) felt lonely was assessed by themselves and their parents using a single item: “I felt lonely./Feels lonely.” (0 = *not true*; 1 = *somewhat or sometimes true*; 2 = *very true or often true*). The item was derived from the respective age-adequate questionnaire of the Achenbach System of Empirically Based Assessment^46^. The response pertains to the last 6 months. Data on emotional loneliness were available from both parents (external assessments – CBCL and Adult Behavior Checklist [ABCL]) and children (self-assessments – YSR and ASR). Values of 1 and 2 were interpreted as indicating experiences of loneliness in the past six months and were dichotomized in the models to represent the presence of loneliness (0 = *no*, 1 = *yes*). We decided to dichotomize the variable because values of 2 were reported by only around 2% of adolescents and 10% of emerging adults, resulting in a sample size too small for reliable statistical modeling.

#### Adverse childhood experiences (FU18)

The German adaptation^47^ of the Adverse Childhood Experiences Questionnaire (ACE-D;^48^) was used to assess ten different types of adverse childhood experiences (e.g., sexual abuse—1 = *yes* or 0 = *no*). The total score (0–10), which is the sum of the individual items, reflects the number of potential traumatic events experienced during childhood and adolescence up to the age of 18. The internal consistency for the ACE-D was α = 0.73 among young adults.

#### School bullying (FU18)

Emerging adults should indicate whether they have ever experienced school bullying or not (0 = *no*, 1 = *yes*).

### Statistical analyses

Usable longitudinal data for the predictors and the outcome variable loneliness were available for 224 emerging adults, 224 mothers, and 173 fathers from the 316 participating families at FU18. Since the data are not assumed to be missing completely at random—as indicated by the dropout analyses—we decided against imputing missing values prior to analyses^49^. These data were included in the subsequent statistical analyses.

To assess the longitudinal stability of loneliness and the agreement between parents and children, Spearman correlation coefficients were calculated. Additionally, an exact McNemar’s test was conducted to examine the stability of loneliness from adolescence to emerging adulthood.

To predict loneliness in adolescence and emerging adulthood, binary logistic regression models were calculated. One model focused on maternal risk factors (maternal mental health problems, maternal dysfunctional parenting, and children’s internalizing and externalizing mental health problems as reported by mothers) assessed during the kindergarten period (at Pre) to predict loneliness in adolescence (at FU10). A second, separate model included the same risk factors from the fathers’ perspectives; however, this model did not reach significance and was therefore not reported. Both models were subsequently extended by adding adolescent risk factors (school bullying, compulsive internet use, and adolescent mental health) as well as ACEs to predict loneliness in emerging adulthood. In this extended model, only adolescent mental health problems emerged as a significant predictor.

This resulted in an additional research question that was added retrospectively –namely, the prediction of adolescent mental health problems as a potential indirect pathway for the prevention of loneliness. Accordingly, multiple linear regression models were calculated to predict adolescent mental health using both childhood and adolescent risk factors, with the aim of identifying further indirect targets for early prevention.

All predictors were entered simultaneously into each model. An examination of variance inflation factors indicated no multicollinearity in any of the logistic and linear regression models (all VIFs < 5).

## Results

### Descriptive analyses and prevalence rates (research question 1)

Descriptive statistics and correlations of the included variables are presented in Table 1. In the descriptive analyses, *loneliness in adolescence* (25.4% felt lonely, *n* = 57) was associated with maternal dysfunctional parenting behavior (*r* = 0.18, *p* = 0.004), compulsive internet use (*r* = 0.22, *p* < 0.001), adolescent mental health problems (*r* = 0.39, *p* < 0.001), and school bullying (*r* = 0.19, *p* = 0.002). *Loneliness in young adulthood* (50.0% felt lonely, *n* = 112) was significantly associated with adverse childhood experiences (*r* = 0.19, *p* = 0.003), compulsive internet use (*r* = 0.19, *p* = 0.003), adolescent mental health problems (*r* = 0.32, *p* < 0.001), and experienced school bullying (*r* = 0.18, *p* = 0.002).

**Table 1:** Descriptive statistics and correlations between mothers, fathers, and children for key study variables.

Variables	1	2	3	4	5	6	7	8	9	10	11	12	13	14	15	16	17	Mean (SD)/in %
1. Triple P	–																	64.6%
2. Migration	.14*	–																18.3%
3. Socioeconomic Status	.06	− .23	–															High = 51.2%
4. Maternal Mental Health Problems (DASS) – Pre	− .01	.04	− .18**	–														23.6 (16.8)
5. Paternal Mental Health Problems (DASS) – Pre	− .04	− .05	− .03	.27***	–													19.4 (14.1)
6. Child Internalizing Mental Health Problems (CBCL- Mothers ) – Pre	.14*	.08	− .19**	.40***	.15**	–												51.1 (9.8)
7. Child Internalizing Mental Health Problems (CBCL- Fathers) – Pre	.05	.06	− .18*	.33***	.35***	.64***	–											51.4 (9.8)
8. Child Externalizing Mental Health Problems (CBCL- Mothers) – Pre	.19**	.12	− .11	.40***	.21***	.66***	.45***	–										50.1 (9.0)
9. Child Externalizing Mental Health Problems (CBCL- Fathers) – Pre	.06	.07	− .09	.30***	.37***	.43***	.69***	.66***	–									51.1 (9.0)
10. Maternal Dysfunctional Parenting (PS) – Pre	.00	.05	− .22***	.41***	.20***	.33***	.28***	.44***	.33***	–								3.2 (0.6)
11. Paternal Dysfunctional Parenting (PS) – Pre	− .01	.08	− .08	.17**	.39***	.16**	.26***	.35***	.38***	.36***	–							3.2 (0.5)
12. Adolescent Adverse Childhood Experiences (ACE-D) – FU18	− .04	.17**	− .25***	.10	.19**	.12*	.09	.01	.12	.11	− .04	–						1.1 (1.7)
13. Adolescent Compulsive Internet Use (CIUS) – FU10	− .01	.05	.01	.05	.00	− .02	− .02	.00	.06	.07	.07	.01	–					25.0 (8.0)
14. Adolescent Mental Health Problems (SDQ) – FU10	.02	.11	− .19**	.20***	.07	.14**	.19**	.23***	.29***	.233***	.09	.24***	.35***	–				9.2 (4.5)
15. Adolescent School Bullying – FU18	− .01	− .01	− .05	.03	.11	.02	− .05	.05	.06	.01	− .07	.21***	.12	.18**	–			58.5%
16. Adolescent Loneliness (YSR) – FU10	− .11	− .01	− .09	.10	.06	.01	.07	− .03	.04	.18**	.01	.10	.22**	.39***	.20**	–		25.4%
17. Emerging Adult Loneliness (ASR) – FU18	− .05	.03	− .01	− .02	.11	.01	− .01	.02	− .02	.03	.10	.19**	.19**	.32***	.18**	.21**	–	50.0%

*p < .05, ** *p* < .01, *** *p* < .001 (two-tailed). Correlations for Triple P, migration background, socioeconomic status, school bullying and the loneliness items were calculated using Spearman correlation coefficients, while all other associations were calculated using Pearson correlation coefficients.

### Research question 2: longitudinal stability of emotional loneliness from adolescence to emerging adulthood

Of the 224 young adults, 38 (16.9%) reported experiencing emotional loneliness at both FU10 and FU18. Of the 57 participants who were lonely at FU10, 38 remained lonely at FU18, which represents two-thirds (66.7%).

For the self-assessments of young adults, a small significant correlation was observed, *r* = 0.21, *p* = 0.002 from adolescence (FU10) to emerging adulthood (FU18). An exact McNemar’s test revealed a statistically significant (Chi^2^ = 31.36, *p* <0.001) change in the proportion of individuals reporting feelings of loneliness from adolescence to emerging adulthood. Similarly, small longitudinal significant correlations were found for the external assessments by mothers (*r* = 0.26, *p* < 0.001; McNemar: Chi^2^ = 4.97, *p* = 0.026) whereas the correlation for fathers (*r* = 0.14, *p* = 0.119; McNemar: Chi^2^ = 0.00; *p* = 1.000) was not significant over the 8-year period.

### Research question 3: parent–child agreement

A small but significant agreement was found between mothers’ (loneliness of adolescents: 9.1% and of emerging adults: 17.6%, according to their mothers) and fathers’ assessment (loneliness of adolescents: 13.3% and of emerging adults: 15.3%, according to their fathers) of the adolescent’s loneliness at FU10 (*r* = 0.28, *p* < 0.001), as well as between mothers and children (*r* = 0.21, *p* = 0.002). However, the father-child agreement was not significant (*r* = 0.12, *p* = 0.108).

In emerging adulthood (FU18), moderate significant correlations were observed between maternal and paternal assessments (*r* = 0.37, *p* < 0.001) and mother–child assessments (*r* = 0.33, *p* < 0.001). The father-child agreement was in the small significant range (*r* = 0.26, *p* = 0.003).

### Research question 4: longitudinal prediction of emotional loneliness from kindergarten age to emerging adulthood

In the first step, logistic regression models were calculated with loneliness in adolescence (FU10) as the outcome. The model including maternal risk factors from early childhood (Pre) is presented in Table 2. It shows that childhood externalizing mental health problems, as reported by mothers, were associated with lower levels of emotional loneliness in adolescence (OR 0.93, *p* = 0.005, 95% CI [0.89, 0.98]), whereas higher levels of maternal dysfunctional parenting behavior were linked to increased loneliness (OR 2.61, *p* = 0.004, 95% CI [1.36, 5.00]). The explained variance of the model was 13% (Nagelkerke’s R^2^).

**Table 2:** Results of the logistic regression model predicting loneliness in adolescence (FU10) based on maternal risk factors in early childhood (Pre).

	Maternal factors				
Reference category: No feelings of loneliness at FU10 – C	*B (SD)*	*p*	Lower (CI 95%)	*OR*	Upper (CI 95%)
Triple P (0 = no; 1 = participation)	− 0.37 (0.33)	.269	0.36	0.69	1.33
Migration background (0 = no; 1 = yes)	0.25 (0.44)	.572	0.54	1.28	3.04
Socioeconomic status (0 = low/middle; 1 = high)	− 0.15 (0.34)	.673	0.44	0.87	1.70
Childhood factors					
Child internalizing mental health problems (CBCL) – M	0.03 (0.02)	.221	0.98	1.03	1.08
Child externalizing mental health problems (CBCL) – M	− 0.07 (0.03)	.005	0.89	0.93	0.98
Maternal mental health problems (DASS) – M	0.01 (0.01)	.237	0.99	1.01	1.03
Maternal dysfunctional parenting (PS) – M	0.96 (0.33)	.004	1.36	2.61	5.00
Constant	− 2.15 (1.29)	.096		0.12	
Model statistics	Chi^2^ (7) = 20.09, *p* = .005; *R*^2^ = .09 (Cox & Snell), .13 (Nagelkerke)				

M indicates values reported by mothers.

In the second step, regression models were calculated to predict loneliness in emerging adulthood (at FU18) (see Table 3). In both the maternal/adolescent (OR 1.19, *p* < 0.001, 95% CI [1.09, 1.30]) and paternal/adolescent (OR 1.20, *p* < 0.001, 95% CI [1.09, 1.33]) models, self-reported adolescent mental health problems emerged as the only significant predictor of loneliness in emerging adulthood. The explained variance of both models was approximately 20%. The factor of school bullying narrowly missed the significance threshold of 5% in both models (Maternal: OR 1.75, *p* = 0.063, 95% CI [0.97, 3.16]; Paternal: OR 1.90, *p* = 0.065, 95% CI [0.96, 3.77]).

**Table 3:** Results of the logistic regression models predicting loneliness in emerging adulthood (FU18) based on maternal, paternal and child risk factors in early childhood and adolescence.

	Maternal/Adolescent model					Paternal/Adolescent model				
Reference category: No feelings of loneliness at FU18 – C	*B (SD)*	*p*	Lower (CI 95%)	*OR*	Upper (CI 95%)	*B (SD)*	*p*	Lower (CI 95%)	*OR*	Upper (CI 95%)
Triple P (0 = no; 1 = participation)	− 0.26 (0.31)	.411	0.42	0.78	1.42	− 0.30 (0.35)	.405	0.37	0.74	1.49
Migration background (0 = no; 1 = yes)	0.05 (0.41)	.899	0.47	1.05	2.37	0.15 (0.48)	.761	0.45	1.16	2.98
Socioeconomic status (0 = low/middle; 1 = high)	0.15 (0.32)	.627	0.63	1.12	2.17	0.14 (0.35)	.700	0.57	1.15	2.29
Adverse childhood experiences (ACE-D) – C	0.14 (0.11)	.206	0.93	1.15	1.42	0.10 (0.14)	.492	0.83	1.10	1.46
Childhood factors										
Child internalizing mental health problems (CBCL) – M/F	− 0.18 (0.02)	.404	0.94	0.98	1.02	− 0.05 (0.03)	.851	0.95	0.99	1.05
Child externalizing mental health problems (CBCL) – M/F	0.01 (0.02)	.827	0.96	1.01	1.05	− 0.04 (0.02)	.150	0.92	0.96	1.01
Parental mental health problems (DASS) – M/F	− 0.01 (0.01)	.887	0.98	0.99	1.02	0.01 (0.01)	.908	0.98	1.00	1.03
Parental dysfunctional parenting (PS) – M/F	0.07 (0.29)	.822	0.61	1.07	1.87	0.49 (0.40)	.227	0.74	1.62	3.57
Adolescent factors										
School bullying – C	0.56 (0.30)	.063	0.97	1.75	3.16	0.64 (0.35)	.065	0.96	1.90	3.77
Compulsive internet use (CIUS) – C	0.01 (0.02)	.950	0.96	1.00	1.04	− 0.02 (0.02)	.469	0.95	0.99	1.03
Adolescent mental health problems (SDQ) – C	0.17 (0.04)	< .001	1.09	1.19	1.30	0.19 (0.05)	< .001	1.09	1.20	1.33
Constant	− 1.58 (1.26)	.209		0.21		− 1.26 (1.59)	.430		0.29	
Model statistics	Chi^2^ (11) = 35.92, *p* < .001; *R*^2^ = .15 (Cox & Snell), .20 (Nagelkerke)					Chi^2^ (11) = 27.07, *p* = .004; *R*^2^ = .15 (Cox & Snell), .19 (Nagelkerke)				

M/F indicates values reported by mothers or fathers and – C indicates values reported by children.

In the third step, multiple linear regression models were calculated to predict adolescent mental health problems at FU10 (see Table 4). In both the maternal/adolescent and paternal/adolescent models, higher levels of reported ACEs (Maternal: β = 0.21, *p* = 0.001; Paternal: β = 0.26, *p* < 0.001) and more compulsive internet use (Maternal: β = 0.30, *p* < 0.001; Paternal: β = 0.27, *p* < 0.001) were significantly associated with greater mental health problems in adolescence. The explained variance ranged between 24% and 26%.

**Table 4:** Results of the multiple linear regression models predicting adolescent mental health problems (FU10) based on maternal, paternal, and child risk factors in early childhood and adolescence.

	Maternal/Adolescent model					Paternal/Adolescent model				
Outcome: Adolescent mental health problems (SDQ) – C	Beta	*p*	Lower (CI 95%)	*B (SD)*	Upper (CI 95%)	Beta	*p*	Lower (CI 95%)	*B (SD)*	Upper (CI 95%)
Triple P (0 = no; 1 = participation)	.03	.622	− 0.77	0.26 (0.52)	6.72	.07	.267	− 0.51	0.66 (0.59)	1.83
Migration background (0 = no; 1 = yes)	.07	.235	− 0.54	0.83 (0.70)	2.20	.03	.709	− 1.31	0.31 (0.82)	1.92
Socioeconomic status (0 = low/middle; 1 = high)	− .09	.160	− 1.82	− 0.76 (0.54)	0.30	− .10	.163	− 2.03	− 0.84 (0.60)	0.34
Adverse childhood experiences (ACE-D) – C	.21	.001	0.23	0.56 (0.17)	0.89	.26	< .001	0.39	0.83 (0.22)	1.26
Childhood factors										
Child internalizing mental health problems (CBCL) – M/F	− .02	.776	− 0.08	− 0.01 (0.04)	0.06	.13	.179	− 0.03	0.06 (0.04)	0.14
Child externalizing mental health problems (CBCL) – M/F	.11	.191	− 0.02	0.05 (0.04)	0.12	.11	.224	− 0.03	0.05 (0.04)	0.13
Parental mental health problems (DASS) – M/F	.12	.075	− 0.01	0.03 (0.02)	0.06	− .03	.732	− 0.05	− 0.01 (0.02)	0.04
Parental dysfunctional parenting (PS) – M/F	.08	.239	− 0.38	0.57 (0.48)	1.52	.08	.286	− 0.50	0.59 (0.56)	1.69
Adolescent factors										
School bullying – C	.08	.182	− 0.32	0.69 (0.51)	1.70	.08	.244	− 0.47	0.68 (0.58)	1.83
Compulsive internet use (CIUS) – C	.30	< .001	0.10	0.16 (0.03)	0.22	.27	< .001	0.07	0.14 (0.03)	0.20
Constant		.582	− 3.78	1.47 (2.66)	6.72		.636	− 7.83	− 1.52 (3.20)	4.80
Model statistics	F (10) = 7.84, *p* < .001; Adjusted *R*^2^ = .24					F (10) = 6.94, *p* < .001; Adjusted *R*^2^ = .26				

M/F indicates values reported by mothers or fathers and – C indicates values reported by children.

None of the three control variables (participation in the Triple P, migration background, and socioeconomic status) showed significant associations with the outcome variables in any of the regression models.

## Discussion

As loneliness among young adults appears to be an increasingly prevalent issue, there is a growing need for both curative and preventive interventions. New strategies should ideally aim to prevent loneliness from developing as early as possible. Within the scope of the “Future Family” project, data from 224 families were analyzed over the course of 18 years to identify early childhood and adolescent factors that are longitudinally associated with the emergence of emotional loneliness in adolescence and in young adulthood.

Our first research question investigated the prevalence of emotional loneliness. The results from our sample indicated that over one-quarter of adolescents reported experiencing emotional loneliness, with this proportion rising to approximately half in young adulthood. This indicates a significant rise in loneliness over an 8-year period. It is important to note that participants reported their experiences over the past six months, suggesting that these findings reflect a more stable phenomenon rather than a transient state. Additionally, data collection at FU18 mostly took place during the COVID-19 pandemic, which likely exacerbated the prevalence of loneliness among young adults^18^. Overall, the observed prevalence rates align with previous findings from Germany (e.g.,^15,16^), although there is notable variability across studies. Despite this variability, emotional loneliness remains a widespread issue among young adults, as supported by the results of our sample. This underscores the importance of early prevention interventions.

Emotional loneliness furthermore appears to be relatively stable over the 8-year study period among those reporting loneliness in adolescence (Research Question 2). This is supported by the small yet significant correlations between child self-reports and maternal assessments. A meta-analysis by Mund et al.^50^ yielded the following results concerning the stability of loneliness: Interindividual differences in loneliness were observed, with some individuals consistently experiencing higher levels of loneliness compared to others, regardless of the current life circumstances. Regarding mean-level development, loneliness generally decreases throughout childhood and remains relatively stable from adolescence into older age. However, some studies suggest a potential increase in loneliness during young adulthood. We still identified a subpopulation comprising 17% of the total sample who reported experiencing (chronic) emotional loneliness during both adolescence and young adulthood. Of the 57 adolescents who reported loneliness, 67% continued to feel lonely in emerging adulthood, indicating a high persistence of emotional loneliness in this specific subsample. This suggests that early experiences of loneliness may be associated with a higher risk of experiencing loneliness later in life, especially during times of crisis such as the COVID-19 pandemic. Future research could focus on identifying the characteristics of those affected, in order to provide timely support to these individuals.

Regarding the agreement between parents and children (Research Question 3), the findings indicate that while parents show moderate correlations with each other, they significantly underestimate the emotional loneliness experienced by their adolescent children. This discrepancy may stem from the tendency of adolescents to distance themselves from their parents during adolescence, sharing less about their emotions. Although this distancing is a normal aspect of adolescent development, it represents a critical opportunity for preventive intervention. By raising parents’ awareness of the importance of discussing emotions with their children, it may be possible to alleviate feelings of emotional loneliness. Interestingly, the level of agreement between parents and their children appears to improve in young adulthood. This increase in concordance could be attributed to the tendency of young adults to reconnect with their parents. Additionally, the COVID-19 pandemic may have contributed to a greater sense of mutual care and more frequent discussions about well-being. Matthews, Fisher et al.^51^ reported a correlation of *r* = 0.34 between sibling and parent ratings of participants’ loneliness and the self-reported loneliness among adolescents/young adults. This correlation is somewhat higher than that observed in our study. This discrepancy may be attributed to the fact that our sample consisted of adolescents with a mean age of 14 years at the time of the FU10 survey, whereas Matthews et al.’s participants were surveyed after the age of 18.

We now turn to our fourth and key research question: To what extent can emotional loneliness in young adulthood be predicted by risk factors present during early childhood (kindergarten age) and adolescence? In the first step, we investigated whether parental mental health problems, child internalizing/externalizing mental health problems, and maternal dysfunctional parenting at kindergarten age were associated with emotional loneliness in adolescence. The maternal models revealed that more child externalizing mental health problems at kindergarten age were associated with lower levels of loneliness during adolescence (FU10). This finding may be explained by the tendency of children with externalizing problems to engage more actively with others, which could alleviate their feelings of loneliness. Thus, early interventions aimed at improving social skills in approaching others and making friends might be beneficial in reducing loneliness later on. Furthermore, maternal dysfunctional parenting at kindergarten age was associated with higher levels of emotional loneliness, while the participation in the Triple P, which was included as a control variable, did not demonstrate a significant effect. Dysfunctional parenting practices in our study were measured using the Parenting Scale^39,40^, which comprises three subscales: laxness, over-reactivity, and hostility. It remains unclear whether one of these dimensions plays a particularly central role, or whether elevated scores across all three subscales are associated with later loneliness. A longitudinal study of high school adolescents found that harsh parenting strategies were linked to increased experiences of loneliness^31^. Further studies suggest that parenting styles involving material rewards and punishments^52^ may contribute to later feelings of loneliness in children. Future research should aim to uncover the mechanisms underlying the association between experienced parenting in childhood and later loneliness.

In the models predicting loneliness in emerging adulthood, only adolescent mental health problems emerged as a significant risk factor. Higher levels of psychological distress during adolescence were associated with an increased likelihood of reporting feelings of loneliness eight years later. This supports previous findings suggesting that loneliness and mental health are significantly interrelated across the developmental course (e.g.,^53,54^). School bullying also emerged as a potential risk factor, showing a trend-level association that narrowly missed statistical significance. However, this finding aligns with existing literature identifying bullying as a relevant risk factor for later loneliness^13,27^.

Based on these findings, we conducted additional analyses to examine whether adolescent mental health problems could be predicted by the selected childhood and adolescent factors. These insights could be used to inform indirect preventive strategies against loneliness. Adolescent mental health problems were significantly associated with the experience of adverse childhood experiences until the age of 18 and compulsive internet use in adolescence, replicating current findings (e.g., ACEs:^55,56^; compulsive internet use^57^:). These factors thus present potential targets for preventive interventions. According to the U.S. Centers for Disease Control and Prevention, ACEs are considered preventable. Various frameworks have been developed in the United States to address and prevent ACEs (e.g.,^58,59^). These frameworks emphasize the importance of strengthening family relationships and recommend intervention and prevention programs. Fortson et al.^59^, for instance, proposed a comprehensive technical package that includes policy, normative, and programmatic activities aimed at preventing ACEs and promoting healthier family environments (e.g., by teaching positive parenting skills). Additionally, promoting engaging activities could serve as a preventive measure against problematic internet use^60^.

Paternal factors did not play a significant role in the prediction of emotional loneliness within our models, underscoring the need to recruit a larger sample of fathers in future studies to study paternal associations. Neither migration background nor SES played a significant role as control variables in our study.

### Strengths and limitations

Our study has several key strengths: First, as an 18-year longitudinal investigation, it provides valuable insights into early prevention strategies that may mitigate the development of emotional loneliness, potentially beginning as early as the kindergarten years. Second, the inclusion of data from mothers, fathers, and children allows for comparisons across these groups and perspectives.

However, the following limitations should also be taken into account: It is important to recognize that the study at hand is based on a relatively small sample of young adults from Germany, which may limit the broader applicability and generalizability of the findings.

Power analyses using G*Power version 3.1^61^ showed that for the logistic regression, a sample size of 420 participants was required to achieve a power of 0.80 with a small effect size (OR 1.44) at the 5% significance level. For linear regression with 10 predictors and small effect sizes, 822 participants were needed. Our sample sizes did not meet these requirements, indicating that the analyses had insufficient power, which may have directly impacted results such as the non-significant effect of bullying as a risk factor. Consequently, the analyses should be interpreted as exploratory. Nonetheless, it was important to include all central risk factors simultaneously in the models to capture their individual and combined effects. Larger samples replicating these findings are necessary. Additionally, future research could analyze these relationships using more complex methods, such as structural equation modeling.

Another important limitation is that the FU18 assessment was carried out during the COVID-19 pandemic, a period when feelings of loneliness markedly increased among young adults^18^, potentially leading to biased outcomes.

Methodologically, loneliness was measured using only one item “I felt lonely” (0 = *not true*; 1 = *somewhat or sometimes true*; 2 = *very true or often true*), with scores of 1 and 2 both considered indicative of emotional loneliness. While this approach may have led to an overestimation of prevalence rates, it was necessitated by the low frequency of the highest response category (“2”) among both adolescents and emerging adults, which made reliable statistical modeling difficult. Ideally, loneliness would have been measured using a validated and multidimensional scale specifically designed for this construct.

## Conclusion

The results of this longitudinal study on emotional loneliness in young adults point to several potential starting points for preventive interventions in childhood and adolescence. These measures range from improving parenting practices and increasing parents’ awareness of behavioral problems in their children during childhood and adolescence, to reducing negative and potentially traumatic childhood experiences, and limiting excessive internet use during adolescence. Given the significant frequency of loneliness in adolescents and especially young adults, it seems advisable to adapt existing prevention programs, to develop new ones, and to systematically investigate their effectiveness.

## Data Availability

The datasets generated and/or analyzed during the current study are not publicly available as they contain sensitive material. Furthermore, it is a longitudinal study with several assessment points. The data could possibly be used to draw conclusions about individuals. The questionnaires used can be found with the corresponding author.
